# Photosystem II Repair and Plant Immunity: Lessons Learned from Arabidopsis Mutant Lacking the THYLAKOID LUMEN PROTEIN 18.3

**DOI:** 10.3389/fpls.2016.00405

**Published:** 2016-03-31

**Authors:** Sari Järvi, Janne Isojärvi, Saijaliisa Kangasjärvi, Jarkko Salojärvi, Fikret Mamedov, Marjaana Suorsa, Eva-Mari Aro

**Affiliations:** ^1^Molecular Plant Biology, Department of Biochemistry, University of TurkuTurku, Finland; ^2^Plant Biology, Department of Biosciences, University of HelsinkiHelsinki, Finland; ^3^Molecular Biomimetics, Department of Chemistry—Ångström Laboratory, Uppsala UniversityUppsala, Sweden

**Keywords:** *Arabidopsis thaliana*, defense, photosynthesis, photosystem II repair cycle, thylakoid lumen, transcriptomics

## Abstract

Chloroplasts play an important role in the cellular sensing of abiotic and biotic stress. Signals originating from photosynthetic light reactions, in the form of redox and pH changes, accumulation of reactive oxygen and electrophile species or stromal metabolites are of key importance in chloroplast retrograde signaling. These signals initiate plant acclimation responses to both abiotic and biotic stresses. To reveal the molecular responses activated by rapid fluctuations in growth light intensity, gene expression analysis was performed with *Arabidopsis thaliana* wild type and the *tlp18.3* mutant plants, the latter showing a stunted growth phenotype under fluctuating light conditions (Biochem. J, 406, 415–425). Expression pattern of genes encoding components of the photosynthetic electron transfer chain did not differ between fluctuating and constant light conditions, neither in wild type nor in *tlp18.3* plants, and the composition of the thylakoid membrane protein complexes likewise remained unchanged. Nevertheless, the fluctuating light conditions repressed in wild-type plants a broad spectrum of genes involved in immune responses, which likely resulted from shade-avoidance responses and their intermixing with hormonal signaling. On the contrary, in the *tlp18.3* mutant plants there was an imperfect repression of defense-related transcripts upon growth under fluctuating light, possibly by signals originating from minor malfunction of the photosystem II (PSII) repair cycle, which directly or indirectly modulated the transcript abundances of genes related to light perception via phytochromes. Consequently, a strong allocation of resources to defense reactions in the *tlp18.3* mutant plants presumably results in the stunted growth phenotype under fluctuating light.

## Introduction

Photosystem II (PSII), embedded in the thylakoid membranes, catalyzes light-dependent water splitting with concomitant oxygen evolution and electron transfer to the plastoquinone pool. PSII consists of the chloroplast-encoded core subunits D1, D2, CP43, and CP47, as well as numerous other subunits, encoded by both the chloroplast and nuclear genomes. Of these proteins, the nuclear-encoded proteins PsbO, PsbP, and PsbQ together with the manganese-calcium cluster form the so called oxygen-evolving complex (OEC), located at the lumenal surface of the PSII complex. In higher plants, the functional PSII complex is formed as a PSII dimer, to which nuclear-encoded light-harvesting complex (LHC) II proteins, Lhcb1-6, are tightly connected forming PSII-LHCII supercomplexes.

Photosynthetic water splitting and evolution of one oxygen molecule require four sequential excitations and subsequent charge separations in the reaction center chlorophyll (Chl) P680, thus producing extremely oxidizing, and potentially hazardous reactive oxygen species (ROS), which enhance oxidative damage to PSII as well as to other thylakoid proteins (Krieger-Liszkay et al., 2008; Pospísil, 2009). Despite the existence of detoxification systems for scavenging of ROS, damage to PSII is unavoidable (Aro et al., 1993; Tyystjärvi and Aro, 1996; Takahashi and Badger, 2011). In particular, the PSII core protein D1 is prone to light-induced damage, and thus an efficient repair cycle has evolved for PSII, which includes proteolytic degradation of damaged D1 protein and its replacement with a newly-synthetized D1 copy (reviewed in Baena-Gonzalez and Aro, 2002; Edelman and Mattoo, 2008; Nixon et al., 2010). These processes involve reversible monomerization of the PSII-LHCII supercomplexes (Danielsson et al., 2006), as well as dynamic changes in grana diameter and in lumen volume (Kirchhoff et al., 2011; Herbstova et al., 2012). A vast number of auxiliary proteins, such as kinases, phosphatases, proteases, transporters, and chaperones have been shown to assist the PSII repair cycle (reviewed in Mulo et al., 2008; Chi et al., 2012; Nickelsen and Rengstl, 2013; Järvi et al., 2015). One of these, the THYLAKOID LUMEN PROTEIN OF 18.3 kDa (TLP18.3) has been shown to be required for efficient degradation of the damaged D1 protein and dimerization of the PSII complex (Sirpiö et al., 2007). Notably, high light treatment challenging the PSII repair cycle triggered only a moderate damage of PSII in *tlp18.3* plants (Sirpiö et al., 2007), which suggest that TLP18.3 is not a crucial component of the repair cycle but instead plays a role in fine tuning the repair cycle. Based on structural data, TLP18.3 has been suggested to be an acidic phosphatase, but only low phosphatase activity was measured for TLP18.3 (Wu et al., 2011). Recently, the regulatory role of the PSII repair cycle has been extended to include the maintenance of photosystem I (PSI) and indeed, insufficient regulation of the PSII repair cycle seems to exert an effect also on the function of PSI (Tikkanen et al., 2014). Moreover, PSII is crucial for plant immunity through production of ROS, which are not only damaging the components of the photosynthetic electron transfer chain, but also act as important retrograde signaling molecules (Rodríguez-Herva et al., 2012; de Torres Zabala et al., 2015). In line with this, a functional connection between PSII repair and regulation of cell death in tobacco leaves infected by tobacco mosaic virus has been established (Seo et al., 2000).

While the exact role of photosynthetic components in sensing and signaling the pathogen infection is only emerging, a wealth of information has accumulated during the past few years on the consequences of fluctuating light on the activity of the photosynthetic machinery (Grieco et al., 2012; Suorsa et al., 2012; Allahverdiyeva et al., 2013; Kono and Terashima, 2014). Nevertheless, we still lack knowledge on how the rapid fluctuations in growth light intensity affect the acclimation processes at the level of nuclear gene expression, and even less is known about potential cross-talk between light acclimation, the PSII repair cycle and disease resistance under fluctuating light. Here, we investigated how the constantly fluctuating growth light intensity modulates the transcript profile of wild-type *Arabidopsis thaliana* (hereafter Arabidopsis) plants, and how such an acclimation response is further affected by the deficiency of the thylakoid lumen protein TLP18.3. Five-week old plants grown either under constant or fluctuating light conditions for their entire life span were used as material to study the late stage of the acclimation process.

## Materials and methods

### Plant material and growth conditions

Arabidopsis, ecotype Columbia 0, wild-type and *tlp18.3* (GABI-Kat 459D12) plants (Sirpiö et al., 2007) were used in all experiments. Plants were grown in 8 h light regime at 23°C either under a photon flux density of 120 μmol photons m^−2^ s^−1^ or under fluctuating light intensities, in which plants were exposed to 50 μmol photons m^−2^ s^−1^ for 5 min and subsequently to high-light of 500 μmol photons m^−2^ s^−1^ for 1 min (Tikkanen et al., 2010), the cycles being repeated during the entire photoperiod. Osram HQI-BT 400 W/D Metal Halide lamps with spectral power distribution from 350 to 800 nm were used as a light source. Five-week-old plants were used for all experiments.

### Gene expression analyses

Microarray analyses of wild-type and *tlp18.3* plants were performed essentially as in Konert et al. (2015). In short, leaf material was harvested 4 h after the onset of the light period in order to be sure that the plants were in a photosynthetically active state and that the PSII repair cycle was properly ongoing and immediately frozen in liquid nitrogen. RNA was isolated using an Agilent Plant RNA isolation mini kit according to manufacturer's instructions. Cy-3 labeled RNA samples were hybridized to Agilent Arabidopsis Gene Expression Microarrays, 4 × 44 K (Design ID 021169) and scanned with Agilent Technologies Scanner G2565CA with a profile AgilentHD _GX_1Color. Numeric data were produced with Agilent Feature Extraction program, version 10.7.3.

Pre-processing of microarrays was performed using Limma's normexp background correction method to avoid negative or zero corrected intensities, followed by between-array normalization using the quantile method to make all array distributions to have the same empirical distribution. Control probes were filtered and then within-array replicate spots were replaced with their average. Pair-wise comparisons between groups were conducted using the Linear Models for Microarray Data (Limma) package Version 3.26.1 from Bioconductor (http://www.bioconductor.org/). The false discovery rate of differentially expressed genes for treatment/control and between-treatment comparisons was based on the Benjamini and Hochberg (BH) procedure. Genes with a score below an adjusted *p*-value threshold of 0.01 and which also showed a minimum of twofold change in expression between conditions or genotype were selected as significantly differentially expressed genes. Gene annotations were obtained from the Arabidopsis Information Resource (TAIR; http://www.arabidopsis.org/). Functional clustering and analysis was performed using the Database for Annotation, Visualization and Integrated Discovery (DAVID) (http://david.abcc.ncifcrf.gov/home.jsp) version 6.7. Differentially expressed genes were compared against gene sets collected from various sources such as publications using the Plant GeneSet Enrichment Analysis Toolkit (PlantGSEA) (http://structuralbiology.cau.edu.cn/PlantGSEA/).

To detect co-regulated gene sets, a cluster analysis of the differentially expressed genes was carried out using data from (Georgii et al., 2012), consisting of microarray data downloaded from NASCArrays (ftp://uiftparabid.nottingham.ac.uk/NASCarrays/By_Experiment_ID/), ArrayExpress (http://www.ebi.ac.uk/microarrayas/ae/), Gene Expression Omnibus (http://www.ncbi.nlm.nih.gov/geo/), and The Integrated Microarray Database System (http://ausubellab.mgh.harvard.edu/). Arrays were normalized with Robust Multi-array Average (RMA), and log2 ratio of the mean of treatment and control expressions across biological replicates was computed. Bayesian Hierarchical Clustering was carried out using R package BHC (Cooke et al., 2011) using log2 fold change ±1 as discretization threshold. Gene set enrichment analysis of the co-regulated gene clusters was carried out using StringDB (http://string-db.org/; Szklarczyk et al., 2015).

### Isolation of the thylakoid membrane and separation of protein complexes

Thylakoid isolation and blue native (BN)-PAGE were performed essentially as described in Järvi et al. (2011). Sodium fluoride was included in thylakoid isolation buffers for samples intended for BN-PAGE, whilst excluded from thylakoids used for spectroscopy analyses (see below). For BN-PAGE, the thylakoid membrane (4 μg Chl) was resuspended into ice-cold 25BTH20G buffer [25 mM BisTris/HCl (pH 7.0), 20% (w/v) glycerol and 0.25 mg ml^−1^ Pefabloc] to a Chl concentration of 1.0 mg ml^−1^. An equal volume of 2.0% (w/v) detergent (n-dodecyl β-D-maltoside, Sigma) solution (diluted in 25BTH20G) was added to the sample and thylakoid membrane was solubilized in darkness for 5 min on ice. Traces of insoluble material were removed by centrifugation at 18,000 g at 4°C for 20 min. Prior to loading, the samples were supplemented with a one-tenth volume of Serva Blue G buffer [100 mM BisTris/HCl (pH 7.0), 0.5 M ACA, 30% (w/v) sucrose, and 50 mg ml^−1^ Serva Blue G].

### Spectroscopic quantitation of PSI and PSII

Room temperature continuous wave electron paramagnetic resonance (EPR) spectroscopy was performed essentially as described in Danielsson et al. (2004) and Suorsa et al. (2015). Measurements were performed at the Chl concentration of 2 mg ml^−1^.

### Photosynthetic activity measurements

The Dual-PAM-100 (Walz, http://www.walz.com/) was used for the measurement of PSII quantum yields. Quantum yields of PSII (F_V_/F_M_, Φ_II_, Φ_NPQ_, and Φ_NO_) were determined from leaves dark adapted for 30 min before the measurements. Saturating pulse (800 ms, 6000 μmol photons m^−2^s^−1^) was applied to determine the maximal fluorescence. Measurements were done in actinic red light of 50, 120, or 500 μmol photons m^−2^s^−1^.

### Statistical analyses

The numerical data were subjected to statistical analysis by Student's *t*-test with statistical significance at the *p* < 0.05.

## Results

### Fluctuating growth light only slightly modified the photosynthetic light reactions

Accumulating evidence during recent years has demonstrated that sudden, abrupt changes in light intensity threaten particularly PSI, not PSII (Grieco et al., 2012; Suorsa et al., 2012; Allahverdiyeva et al., 2013; Kono and Terashima, 2014). Indeed, quantitation of the functional PSI/PSII ratios from wild-type plants with EPR revealed a PSI/PSII ratio of 1.12 for plants grown under constant light conditions (Suorsa et al., 2015), whereas plants grown under fluctuating light conditions exhibited a clearly lower value, 1.02.

**Figure 1:**
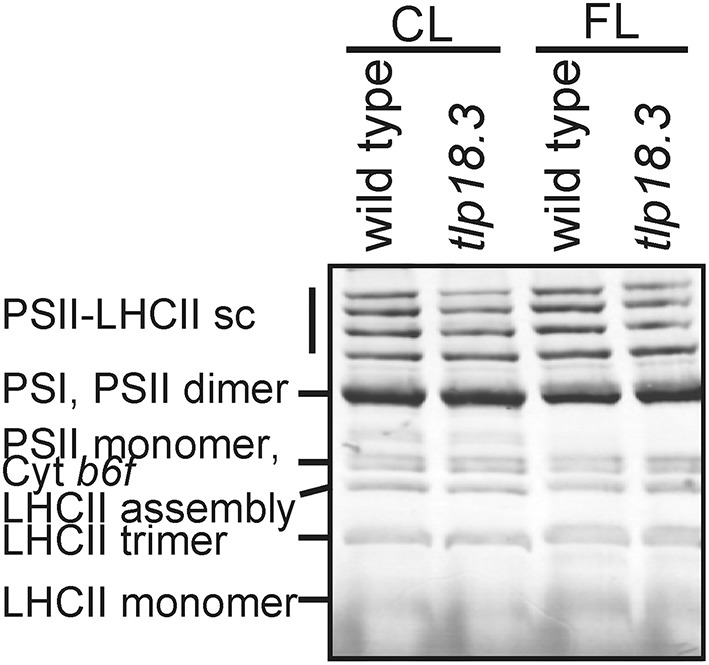
**Accumulation of thylakoid protein complexes in wild-type and *tlp18.3* plants**. Plants were grown in 8 h light regime either in a photon flux density of 120 μmol photons m^−2^ s^−1^ (constant growth light; CL) or 50 μmol photons m^−2^s^−1^ for 5 min and 500 μmol μmol photons m^−2^s^−1^ for 1 min (FL, fluctuating light). sc. supercomplex. A representative example from three independent biological replications is shown.

The *tlp18.3* plants showed a distinct stunted phenotype upon growth under fluctuating white light and the dry weight of the *tlp18.3* plants (12.2 ± 5.7 mg) was markedly decreased as compared to wild type (29.9 ± 4.7 mg; *n* = 6). This observation prompted us to monitor whether the oligomeric structure of the thylakoid membrane protein complexes of wild-type and *tlp18.3* plants grown either under constant or fluctuating light conditions is altered. Malfunction of the PSII repair cycle is often evidenced by a low amount of the most active PSII complexes, the PSII-LHCII complexes, accompanied by a high amount of PSII monomers, which are under the repair cycle (Danielsson et al., 2006). To that end, the BN-PAGE separation of thylakoid protein complexes according to their molecular mass was applied. In line with earlier results (Sirpiö et al., 2007), the *tlp18.3* thylakoids accumulated slightly less of the PSII-LHCII complexes under constant light (Figure 1). Similar result was also evident under fluctuating light intensities, the amount of PSII-LHCII being somewhat lower in *tlp18.3* plants as compared to wild type. However, no significant differences were observed in heterogeneity of the photosynthetic protein complexes, when wild-type and mutant plants grown either under constant or fluctuating light were compared (Figure 1). A previous report has shown that the maximal PSII quantum yield is not changed in *tlp18.3* plants grown under constant growth light conditions as compared to wild type (Sirpiö et al., 2007). In line with this, the maximum quantum yield and effective quantum yields of PSII remained rather similar, when the *tlp18.3* and wild-type plants grown their entire life span under fluctuating light were compared (Table 1). Indeed, the PSII activity was only slightly down-regulated in *tlp18.3* plants as compared to wild type. Thus, the growth defect shown by the *tlp18.3* plants under fluctuating light intensities does not originate from the diminished pool of active PSII complexes.

**Table 1:** **PSII quantum yields of wild-type and *tlp18.3* plants grown under fluctuating light**.

**Photosynthetic parameter**	**Wild type**	***tlp18.3***
**EFFECTIVE PSII QUANTUM YIELD, Φ_I*I*_**		
50 μmol photons m^−2^s^−1^	0.50 ± 0.02	0.47 ± 0.04
120 μmol photons m^−2^s^−1^	0.28 ± 0.06	0.26 ± 0.03
500 μmol photons m^−2^s^−1^	0.04 ± 0.01	0.03 ± 0.01
**NON-PHOTOCHEMICAL ENERGY DISSIPATION, Φ_NPQ_**		
50 μmol photons m^−2^s^−1^	0.13 ± 0.02	0.15 ± 0.04
120 μmol photons m^−2^s^−1^	0.48 ± 0.07	0.47 ± 0.03
500 μmol photons m^−2^s^−1^	0.68 ± 0.01	0.66 ± 0.01^*^
**YIELD OF NON-REGULATED NON-PHOTOCHEMICAL ENERGY LOST, Φ_NO_**		
50 μmol photons m^−2^s^−1^	0.37 ± 0.01	0.38 ± 0.03
120 μmol photons m^−2^s^−1^	0.24 ± 0.01	0.27 ± 0.00^*^
500 μmol photons m^−2^s^−1^	0.28 ± 0.00	0.31 ± 0.02
**MAXIMAL QUANTUM YIELD OF PSII, F_V_/F_M_**		
	0.78 ± 0.01	0.76 ± 0.02^*^

The values are the means ± SD,n = 4–5, except for F_V_/F_M_ n = 12. Statistically significant differences comparing the mutant plants to that of the corresponding wild type are marked with asterix (

* *). See text for details*.

### Consequences of fluctuating growth light intensity on gene expression

To further characterize plant acclimation to fluctuating light, we performed transcript profiling of the wild-type and *tlp18.3* plants grown under constant and fluctuating light intensities and compared the four datasets: (*i*) wild-type plants grown under fluctuating vs. constant growth light, (*ii*) *tlp18.3* plants grown under fluctuating vs. constant growth light, (*iii*) *tlp18.3* vs. wild-type plants grown under fluctuating light, and (*iv*) *tlp18.3* vs. wild-type plants grown under constant light. Gene enrichment analysis and functional annotation clustering of differentially expressed genes were performed using the DAVID bioinformatic resource (the cutoff was set to logFC > 1 and the adjusted *p*-value threshold to a minimum of 0.01).

**Figure 2:**
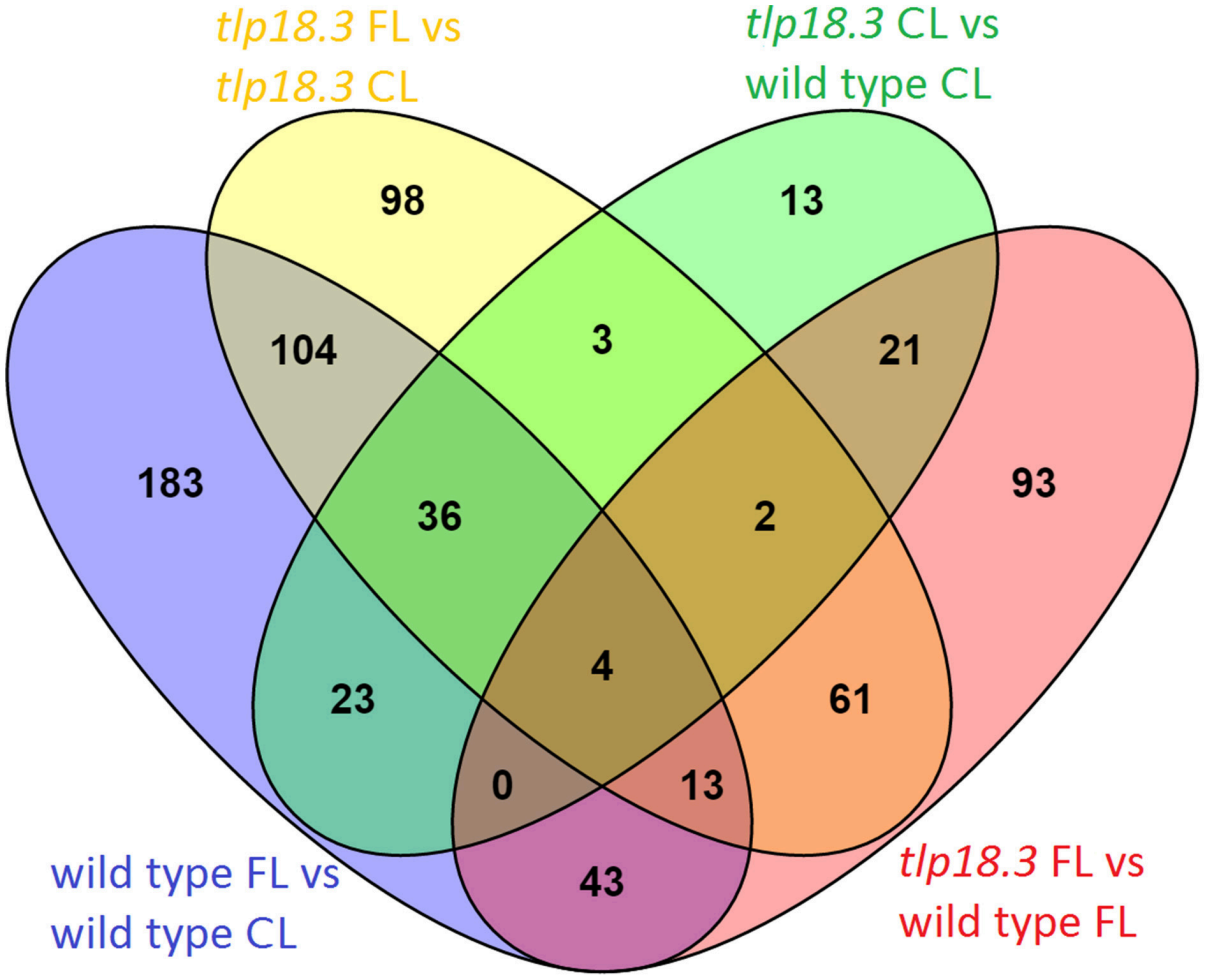
**Venn diagram showing the overlap of significantly differentially regulated genes (logFC > 1) in response to either fluctuating light (FL) as compared to constant growth light (CL) or deficient function of the TLP18.3 protein**.

Wild-type plants grown under fluctuating light showed significantly different transcript abundance for 406 genes as compared to wild type grown under constant light, whereas in the *tlp18.3* mutant, 321 genes responded differentially to fluctuating light as compared to growth light (Figure 2). When the transcript abundances between the genotypes was compared, 237 genes showed significantly different transcript abundance in *tlp18.3* plants compared to wild type when grown under fluctuating light conditions, whereas under constant growth light the number of differentially expressed genes between wild type and the *tlp18.3* mutant was 102 (Figure 2). Thus, it can be concluded that the growth light condition altered the number of differentially regulated genes more pronouncedly than the genotype. Moreover, the wild-type plants showed more profound changes at their gene expression level as a response to fluctuating growth light than the *tlp18.3* plants.

#### Plants grown under fluctuating light did not show differential abundance of photosynthesis related transcripts

Examination of differentially expressed genes revealed no photosynthesis-related gene ontologies in any of the four datasets analyzed (Tables 2, 3). Indeed, no gene ontologies related to photosynthetic light reactions, Calvin-Benson-Bassham cycle, or biosynthesis of photosynthetic pigments was observed in the gene enrichment analysis. Presumably, regulation of the photosynthetic machinery at transcriptional level does not play an important role during acclimation to relatively mild light intensity fluctuations, being designed such that the total amount of photons hitting the leaf remained nearly unchanged during the 8 h light period, when constant and fluctuating light conditions were compared. Likewise, deficient function of the TLP18.3 protein had only minor effects on transcript abundance of various photosynthesis genes.

**Table 2:** **Classification of significantly differently expressed genes base on gene enrichment analysis of plants grown either under fluctuating light (FL) or constant growth light (CL): (A) Gene enrichment analysis of wild-type plants grown either under fluctuating or constant light; (B) Gene enrichment analysis of *tlp18.3* plants grown either under fluctuating or constant light**.

**Term**		**Count**	***P*-value**
**(A) WILD TYPE FL vs. WILD TYPE CL**			
**Increased Transcript Abundance**			
GOTERM_MF_FAT	GO:0005507 copper ion binding	5	0.0055
GOTERM_CC_FAT	GO:0031225 anchored to membrane	6	0.0076
**Decreased Transcript Abundance**			
GOTERM_BP_FAT	GO:0006952 defense response	43	3.26E-14
GOTERM_MF_FAT	GO:0004672 *protein kinase activity*	40	7.91E-12
GOTERM_BP_FAT	GO:0010033 *response to organic substance*	42	1.18E-11
GOTERM_BP_FAT	GO:0006468 *protein amino acid phosphorylation*	39	4.18E-11
GOTERM_BP_FAT	GO:0009751 *response to salicylic acid stimulus*	16	8.69E-11
GOTERM_BP_FAT	GO:0006955 immune response	20	4.62E-10
GOTERM_BP_FAT	GO:0016310 *phosphorylation*	39	7.75E-10
GOTERM_BP_FAT	GO:0010200 response to chitin	14	1.24E-09
GOTERM_MF_FAT	GO:0004674 *protein serine/threonine kinase activity*	33	4.75E-09
GOTERM_BP_FAT	GO:0006796 *phosphate metabolic process*	39	6.76E-09
GOTERM_BP_FAT	GO:0006793 *phosphorus metabolic process*	39	6.91E-09
GOTERM_BP_FAT	GO:0045087 innate immune response	18	8.31E-09
GOTERM_BP_FAT	GO:0009617 *response to bacterium*	17	1.02E-08
GOTERM_BP_FAT	GO:0009611 *response to wounding*	13	7.11E-08
GOTERM_BP_FAT	GO:0042742 *defense response to bacterium*	14	1.10E-07
GOTERM_BP_FAT	GO:0009743 response to carbohydrate stimulus	14	2.74E-07
GOTERM_MF_FAT	GO:0032559 *adenyl ribonucleotide binding*	49	1.85E-06
GOTERM_MF_FAT	GO:0030554 *adenyl nucleotide binding*	50	5.06E-06
GOTERM_MF_FAT	GO:0001883 *purine nucleoside binding*	50	5.06E-06
GOTERM_MF_FAT	GO:0001882 *nucleoside binding*	50	5.54E-06
GOTERM_MF_FAT	GO:0005524 ATP binding	47	7.74E-06
GOTERM_BP_FAT	GO:0009814 defense response, incompatible interaction	9	9.97E-06
GOTERM_BP_FAT	GO:0009873 ethylene mediated signaling pathway	11	1.73E-05
GOTERM_BP_FAT	GO:0009723 response to ethylene stimulus	13	2.44E-05
GOTERM_MF_FAT	GO:0032555 purine ribonucleotide binding	49	3.11E-05
GOTERM_MF_FAT	GO:0032553 ribonucleotide binding	49	3.11E-05
**(A) WILD TYPE FL vs. WILD TYPE CL**			
GOTERM_BP_FAT	GO:0009753 response to jasmonic acid stimulus	10	5.35E-05
GOTERM_BP_FAT	GO:0009719 response to endogenous stimulus	26	5.38E-05
GOTERM_MF_FAT	GO:0017076 purine nucleotide binding	50	7.16E-05
GOTERM_BP_FAT	GO:0000160 two-component signal transduction system	11	1.41E-04
GOTERM_MF_FAT	GO:0005529 sugar binding	8	3.13E-04
GOTERM_MF_FAT	GO:0000166 nucleotide binding	52	0.0016
GOTERM_MF_FAT	GO:0004713 protein tyrosine kinase activity	11	0.0016
GOTERM_BP_FAT	GO:0009725 response to hormone stimulus	21	0.0021
GOTERM_BP_FAT	GO:0009816 defense response to bacterium	4	0.0028
GOTERM_BP_FAT	GO:0009620 *response to fungus*	13	0.0031
GOTERM_MF_FAT	GO:0005509 calcium ion binding	12	0.0034
GOTERM_BP_FAT	GO:0009863 salicylic acid mediated signaling pathway	4	0.0038
GOTERM_BP_FAT	GO:0006979 response to oxidative stress	10	0.0043
GOTERM_BP_FAT	GO:0043900 regulation of multi-organism process	3	0.0050
GOTERM_CC_FAT	GO:0005618 cell wall	15	0.0057
GOTERM_BP_FAT	GO:0009867 jasmonic acid mediated signaling pathway	4	0.0065
GOTERM_CC_FAT	GO:0030312 external encapsulating structure	15	0.0065
GOTERM_BP_FAT	GO:0016265 death	9	0.0068
GOTERM_BP_FAT	GO:0008219 cell death	9	0.0068
GOTERM_CC_FAT	GO:0012505 endomembrane system	59	0.0073
GOTERM_MF_FAT	GO:0030246 carbohydrate binding	8	0.0073
GOTERM_BP_FAT	GO:0009625 response to insect	3	0.0099
**(B) *tlp18.3* FL vs. *tlp18.3* CL**			
**Increased Transcript Abundance**			
GOTERM_BP_FAT	GO:0009611 *response to wounding*	8	1.33E-04
GOTERM_BP_FAT	GO:0010224 response to UV-B	5	4.47E-04
GOTERM_MF_FAT	GO:0080030 methyl indole-3-acetate esterase activity	3	0.0013
GOTERM_BP_FAT	GO:0009628 response to abiotic stimulus	20	0.0017
GOTERM_BP_FAT	GO:0009411 response to UV	5	0.0022
GOTERM_MF_FAT	GO:0030414 peptidase inhibitor activity	4	0.0032
GOTERM_BP_FAT	GO:0009620 *response to fungus*	10	0.0064
GOTERM_MF_FAT	GO:0004857 enzyme inhibitor activity	6	0.0081
GOTERM_BP_FAT	GO:0009416 response to light stimulus	10	0.0094
**(B) *tlp18.3* FL vs. *tlp18.3* CL**			
GOTERM_MF_FAT	GO:0005385 zinc ion transmembrane transporter activity	3	0.0099
**Decreased Transcript Abundance**			
GOTERM_BP_FAT	GO:0009751 *response to salicylic acid stimulus*	8	4.23E-06
GOTERM_BP_FAT	GO:0009617 *response to bacterium*	9	1.14E-05
GOTERM_MF_FAT	GO:0004672 *protein kinase activity*	15	8.88E-05
GOTERM_MF_FAT	GO:0004674 *protein serine/threonine kinase activity*	13	3.89E-04
GOTERM_BP_FAT	GO:0006468 *protein amino acid phosphorylation*	14	6.61E-04
GOTERM_BP_FAT	GO:0006793 *phosphorus metabolic process*	15	0.0011
GOTERM_BP_FAT	GO:0042742 *defense response to bacterium*	6	0.0013
GOTERM_BP_FAT	GO:0016310 *phosphorylation*	14	0.0017
GOTERM_BP_FAT	GO:0006796 *phosphate metabolic process*	14	0.0033
GOTERM_BP_FAT	GO:0006869 lipid transport	5	0.0041
GOTERM_BP_FAT	GO:0010033 *response to organic substance*	13	0.0049
GOTERM_BP_FAT	GO:0010876 lipid localization	5	0.0061
GOTERM_MF_FAT	GO:0030554 *adenyl nucleotide binding*	19	0.0078
GOTERM_MF_FAT	GO:0001883 *purine nucleoside binding*	19	0.0078
GOTERM_MF_FAT	GO:0001882 *nucleoside binding*	19	0.0081
GOTERM_MF_FAT	GO:0032559 *adenyl ribonucleotide binding*	18	0.0092

*Categories, which co-exist in (A) and (B), are italicized*.

*Gene enrichment analysis was performed using DAVID (adjusted p-value threshold minimum 0.01). % indicates the percentage of genes differentially regulated over the number of total genes within the term. BP, biological process; CC, cellular component; GO, gene ontology; MF, molecular function*.

**Table 3:** **Classification of significantly differentially expressed genes base on gene enrichment analysis in wild-type and *tlp18.3* plants: (A) Gene enrichment analysis of in *tlp18.3* plants as compared to wild-type plants grown under fluctuating light (FL); (B) Gene enrichment analysis of in *tlp18.3* plants as compared to wild-type plants grown under constant light (CL)**.

**Term**		**Count**	***P*-value**
**(A) *tlp18.3* FL vs. WILD TYPE FL**			
**Increased Transcript Abundance**			
GOTERM_BP_FAT	GO:0009611 response to wounding	12	1.75E-10
GOTERM_BP_FAT	GO:0010033 response to organic substance	24	7.66E-09
GOTERM_BP_FAT	GO:0010200 response to chitin	10	1.45E-08
GOTERM_BP_FAT	GO:0009743 response to carbohydrate stimulus	11	5.85E-08
GOTERM_BP_FAT	GO:0009719 response to endogenous stimulus	18	5.33E-06
GOTERM_BP_FAT	GO:0009725 response to hormone stimulus	16	4.05E-05
GOTERM_BP_FAT	GO:0009723 response to ethylene stimulus	9	4.41E-05
GOTERM_BP_FAT	GO:0006952 defense response	16	1.66E-04
GOTERM_BP_FAT	GO:0000160 two-component signal transduction system	7	8.21E-04
GOTERM_BP_FAT	GO:0009628 response to abiotic stimulus	16	8.28E-04
GOTERM_BP_FAT	GO:0009409 response to cold	7	0.0012
GOTERM_BP_FAT	GO:0009873 ethylene mediated signaling pathway	6	0.0017
GOTERM_BP_FAT	GO:0009612 response to mechanical stimulus	3	0.0029
GOTERM_BP_FAT	GO:0009631 cold acclimation	3	0.0045
GOTERM_BP_FAT	GO:0006869 lipid transport	5	0.0066
GOTERM_BP_FAT	GO:0009620 response to fungus	8	0.0066
GOTERM_CC_FAT	GO:0012505 endomembrane system	29	0.0072
GOTERM_BP_FAT	GO:0009753 response to jasmonic acid stimulus	5	0.0081
GOTERM_BP_FAT	GO:0009266 response to temperature stimulus	7	0.0090
GOTERM_BP_FAT	GO:0010876 lipid localization	5	0.0098
**Decreased Transcript Abundance**			
GOTERM_BP_FAT	GO:0009642 response to light intensity	5	5.96E-05
GOTERM_BP_FAT	GO:0006979 response to oxidative stress	7	1.73E-04
GOTERM_MF_FAT	GO:0004784 superoxide dismutase activity	3	2.66E-04
GOTERM_MF_FAT	GO:0016721 oxidoreductase activity.	3	2.66E-04
GOTERM_BP_FAT	GO:0009628 response to abiotic stimulus	12	4.88E-04
GOTERM_BP_FAT	GO:0000302 response to reactive oxygen species	5	7.28E-04
GOTERM_BP_FAT	GO:0006801 superoxide metabolic process	3	7.45E-04
GOTERM_BP_FAT	GO:0010035 response to inorganic substance	8	8.78E-04
**(A) *tlp18.3* FL vs. WILD TYPE FL**			
GOTERM_MF_FAT	GO:0005507 copper ion binding	5	0.0013
GOTERM_BP_FAT	GO:0009416 response to light stimulus	7	0.0022
GOTERM_BP_FAT	GO:0009314 response to radiation	7	0.0026
GOTERM_BP_FAT	GO:0009617 response to bacterium	5	0.0055
GOTERM_BP_FAT	GO:0009063 cellular amino acid catabolic process	3	0.0073
GOTERM_BP_FAT	GO:0009644 response to high-light intensity	3	0.0073
GOTERM_BP_FAT	GO:0009310 amine catabolic process	3	0.0083
**(B) *tlp18.3* CL vs. WILD TYPE CL**			
**Increased Transcript Abundance**			
GOTERM_MF_FAT	GO:0030614 oxidoreductase activity.	5	1.92E-09
GOTERM_MF_FAT	GO:0008794 arsenate reductase (glutaredoxin) activity	5	1.92E-09
GOTERM_MF_FAT	GO:0030613 oxidoreductase activity.	5	1.92E-09
GOTERM_MF_FAT	GO:0030611 arsenate reductase activity	5	2.62E-09
GOTERM_MF_FAT	GO:0015035 protein disulfide oxidoreductase activity	6	5.97E-09
GOTERM_MF_FAT	GO:0015036 disulfide oxidoreductase activity	6	1.21E-08
GOTERM_MF_FAT	GO:0016667 oxidoreductase activity	6	1.84E-07
GOTERM_BP_FAT	GO:0045454 cell redox homeostasis	6	8.27E-07
GOTERM_BP_FAT	GO:0022900 electron transport chain	6	2.05E-06
GOTERM_BP_FAT	GO:0019725 cellular homeostasis	6	8.08E-06
GOTERM_BP_FAT	GO:0042592 homeostatic process	6	2.07E-05
GOTERM_BP_FAT	GO:0006091 generation of precursor metabolites and energy	6	1.23E-04
GOTERM_MF_FAT	GO:0009055 electron carrier activity	6	0.0012
**Decreased Transcript Abundance**			
GOTERM_BP_FAT	GO:0009751 response to salicylic acid stimulus	5	4.07E-04
GOTERM_MF_FAT	GO:0004672 protein kinase activity	8	0.0038
GOTERM_BP_FAT	GO:0010033 response to organic substance	9	0.0050
GOTERM_MF_FAT	GO:0004674 protein serine/threonine kinase activity	7	0.0086

*Gene enrichment analysis was performed using DAVID (adjusted p-value threshold minimum 0.01). % indicates the percentage of genes differentially regulated over the number of total genes within the term. BP, biological process; CC, cellular component; GO, gene ontology; MF, molecular function*.

#### Fluctuating light conditions induced transcriptional adjustments in immunity related genes both in wild-type and *tlp18.3* plants

Bioinformatic analysis revealed that the majority of differentially expressed gene ontologies between plants grown under fluctuating and constant light conditions were linked to biotic or abiotic stress responses (Tables 2A,B). In wild type, growth under fluctuating light resulted in decreased transcript abundance within numerous gene ontologies related to plant immunity, as compared to wild type grown under constant light (Table 2A). These genes included mitogen-activated protein kinases (MAPKs) involved in early defense signaling, Toll/Interleukin-1 receptor-nucleotide binding site (TIR-NBS) class resistance (R) proteins mediating effector-triggered immunity (ETI) as well as pathogen related defense proteins, such as plant defensins (Supplementary Table 1). In contrast, the *tlp18.3* mutant showed both decreased and increased transcript abundance within gene ontologies related to plant immunity, when fluctuating and constant light grown plants were compared to each other (Table 2B). For example, ankyrin *BDA1* (*AT5G54610*), which is induced by salicylic acid (SA) and is involved in innate immunity (Blanco et al., 2005; Yang et al., 2012) showed cumulative repression in the transcript abundance in response to fluctuating light and deficient function of the TLP18.3 protein. In contrast, plant defensin *PDF2.1* (*AT2G02120*) and defensin-like (*AT2G43535*) genes, which are activated in response to fungal infection, were induced in *tlp18.3* plants under fluctuating light.

With respect to abiotic stress, gene ontologies “response to UV” and “response to light stimulus” were enriched in the transcriptome of *tlp18.3* leaves, when plants grown under fluctuating and constant light were compared (Table 2B). For example, increased abundance of transcripts for *EARLY LIGHT-INDUCED PROTEIN2* (*ELIP2*; *AT4G14690*), which modulates Chl biosynthesis to prevent photo-oxidative stress (Tzvetkova-Chevolleau et al., 2007; Hayami et al., 2015), was observed in the fluctuating-light-grown *tlp18.3* plants (Supplementary Table 1). In contrast, no gene ontologies related to light perception showed differential expression in the wild-type plants as a response to fluctuating light (Table 2A). Decreased transcript abundance of gene ontologies associated with lipid localization and lipid transport were also observed as response to fluctuating light specifically in *tlp18.3* leaves. Several genes encoding lipid-transfer proteins such as *LIPID TRANSFER PROTEIN 3* (*LTP3*; *AT5G59320*), which mediates freezing and drought stress in Arabidopsis (Guo et al., 2013), were down-regulated in the *tlp18.3* mutant, when plants were grown under fluctuating light as compared to constant growth light (Supplementary Table 1).

When fluctuating-light-grown *tlp18.3* and wild-type plants were compared to each other, increased transcript abundance of genes related to the defense mechanisms in the *tlp18.3* mutant was again the most prominent result (Table 3A). Enrichment analysis and functional annotation clustering of the differentially expressed gene ontologies in *tlp18.3* and wild-type plants also revealed that several gene clusters related to abiotic stresses were differentially expressed in *tlp18.3* plants as compared to wild type under fluctuating light. Decreased transcript abundance of gene ontologies “response to light stimulus” and “response to oxidative stress” was observed in the *tlp18.3* mutant as compared to wild type. Closer look at the genes among these categories pinpointed that the transcript abundance for cytosolic and chloroplastic *COPPER/ZINC SUPEROXIDE DISMUTASES 1* (*AT1G08830*) and 2 (*AT2G28190*), respectively, was repressed in *tlp18.3* plants as compared to wild type under fluctuating light conditions (Supplementary Table 1).

Finally, when constant-light-grown *tlp18.3* and wild-type plants were compared, only a few gene ontologies related to biotic or abiotic stresses were identified (Table 3B). This result is consistent with the postulated role of TLP18.3 specifically during the dynamic light acclimation process, as evidenced by the distinct growth phenotype of the mutant plants under fluctuating light.

#### Adjustments in immunity-related genes under fluctuating light are linked to plant hormones

Plant acclimation to various stresses, including light stress, is regulated by signaling cascades, which include plant hormones as central components (Karpinski et al., 2013; Müller and Munné-Bosch, 2015). In wild-type plants, growth under fluctuating light as compared to constant light resulted in reduced transcript abundance of several genes related to SA signaling cascades (Table 2A). For example, expression of a gene encoding SYSTEMIC ACQUIRED RESISTANCE DEFICIENT 1 (SARD1; AT1G73805), a key regulator of ISOCHORISMATE SYNTHASE 1, a rate-limiting enzyme in pathogen-induced SA biosynthesis (Zhang et al., 2010), was shown to be down-regulated in wild-type plants grown under fluctuating light. Also expression of a gene encoding BENZOIC ACID/SA CARBOXYL METHYLTRANSFERASE 1 (BSMT1; AT3G11480), which synthetizes methyl salicylate (a mobile signal molecule for plant systemic acquired resistance) from SA (Park et al., 2007), was down-regulated in fluctuating light. In line with these results, *WALL-ASSOCIATED KINASE 2* (*WAK2; AT1G21270*) and *L-TYPE LECTIN RECEPTOR KINASE IV.1* (*LecRK-IV.1; AT2G37710*), which are both induced by SA, showed reduced transcript abundance in wild-type plants as response to fluctuating light (He et al., 1999; Blanco et al., 2005) (Supplementary Table 1). Also the *tlp18.3* plants grown under fluctuating light showed decreased abundance of gene transcripts related to SA signaling as compared to plants grown under constant light (Table 2B). However, the number of repressed genes was lower in the *tlp18.3* mutant as compared to wild type and no differential expression of SARD1 or BSMT1 were observed in *tlp18.3* plants as response to fluctuating light (Table 2, Supplementary Table 1). Decreased amount of transcripts related to SA signaling was also evident when *tlp18.3* plants grown under constant light were compared to wild type (Table 3B), while no difference in SA signaling was observed between *tlp18.3* and wild-type plants grown under fluctuating light (Table 3A). To that end, the fluctuating light condition and to a lesser extent deficient function of the TLP18.3 protein repressed the SA responsive genes.

Similarly, ethylene (ET)- and jasmonate (JA)-related defense pathways showed reduced transcript abundance in wild-type plants grown under fluctuating light as compared to constant light (Table 2A), while in the *tlp18.3* mutant no difference was observed in ET/JA defense reactions between the light conditions (Table 2B). It seems that the repression of ET/JA responsive gene expression under fluctuating light is blocked in the *tlp18.3* mutants, which became apparent when ET/JA responses between fluctuating light grown *tlp18.3* and wild-type plants were compared (Table 3A).

The most prominent alteration in the gene ontology level, when the transcript abundances of constant light grown *tlp18.3* and wild-type plants were compared, was an increase in transcripts of six genes encoding CC-type glutaredoxins (*ROXY 5, ROXY 11-15*) and two of those, *ROXY 5* and *ROXY 13*, were up-regulated in *tlp18.3* as compared to wild type also under fluctuating light (Tables 3, 4, Supplementary Table 1). As CC-type glutaredoxins have been suggested to be capable of suppressing the JA and ET-induced defense genes (Zander et al., 2012), a causal connection might exist between expression of JA and ET-responsive genes and differential expression of *ROXY* genes. It can be concluded that alteration in the gene expression patterns of SA, ET, and JA signaling are taking place during plant acclimation to fluctuating light and that these alterations are strongly affected by the deficient function of the TLP18.3 protein.

**Table 4:** **List of genes which are significantly differentially expressed in *tlp18.3* plants as compared to wild type both under fluctuating (FL) and constant light (CL) conditions (logFC > 1)**.

**Gene**		**logFC FL**	**logFC CL**
Drought-repressed 4	AT1G73330	2.06	1.15
ELF4	AT2G40080	1.72	1.60
Major facilitator superfamily protein	AT5G62730	1.46	1.25
Major facilitator superfamily protein	AT2G16660	1.32	1.18
Monothiol glutaredoxin-S4/ROXY 13	AT4G15680	1.21	1.57
Putative glutaredoxin-C12/ROXY 5	AT2G47870	1.18	1.23
Delta-9 acyl-lipid desaturase 1	AT1G06080	−1.35	−1.01
HAD superfamily, subfamily IIIB acid phosphatase	AT4G29270	−1.94	−1.54
Transcription factor PIL1	AT2G46970	−2.23	−1.37
Transcription factor HFR1	AT1G02340	−2.31	−1.29
TLP18.3	AT1G54780	−7.13	−7.07

#### Phytochrome-mediated light signaling is likely to be altered in *tlp18.3* plants

Next, we wanted to further explore which Arabidopsis genes showed a differential expression pattern in the *tlp18.3* plants both under constant and fluctuating light conditions. In addition to ROXY5 and ROXY13 located in the endomembrane system, genes encoding cold (DELTA-9 DESATURASE 1) and drought-repressed (DROUGHT-REPRESSED 4) proteins, acid phosphatase (AT4G29270), and two putative membrane transporters (AT5G62730, AT2G16660) showed differential expression in the *tlp18.3* mutant. Interestingly, two genes encoding bHLH class phytochrome A-signaling components, LONG HYPOCOTYL IN FAR-RED 1 (HFR1; AT1G02340) and PHYTOCHROME INTERACTING FACTOR 3-LIKE 1 (PIL1; AT2G46970; Fairchild et al., 2000; Salter et al., 2003), showed decreased transcript abundance in *tlp18.3* plants as compared to wild type (Table 4). Instead, expression of the gene encoding EARLY FLOWERING 4 (ELF4; AT2G40080), a phytochrome-controlled regulator of circadian clock was induced in the *tlp18.3* mutant as compared to wild type. Taken together, the deficient function of TLP18.3 is likely to change the phytochrome-mediated light signaling both under constant and fluctuating light intensities.

#### Decreased transcript abundance of dark-induced genes suggest that nitrogen to carbon and/or phosphorus to carbon ratios might be altered in *tlp18.3* plants under fluctuating light

Nutrient availability plays an important regulatory role in growth and development of plants, but also cross-talk between nutrient availability and disease resistance exist (Huber, 1980; Hermans et al., 2006). Interestingly, *GLUTAMINE-DEPENDENT ASPARAGINE SYNTHASE 1*/*DARK-INDUCED 6* (*ASN1/DIN6; AT3G47340)* and *DARK-INDUCED 1*/*SENESCENCE 1* (*DIN1/SEN1; AT4G35770*) genes showed strong down-regulation in fluctuating light grown *tlp18.3* plants as compared to either fluctuating light grown wild type or constant light grown *tlp18.3* plants (Supplementary Table 1). ASN1/DIN6 regulates the flow of nitrogen into asparagine, which acts as a nitrogen storage and transport compound in darkness and its gene expression is regulated by the nitrogen to carbon ratio (Lam et al., 1994). DIN1/SEN1, which has been suggested to contribute to enhanced susceptibility to plant viruses, is induced by phosphate starvation and repressed by sugars (Fernández-Calvino et al., 2015). The differential expression of *ASN1/DIN6* and *DIN1/SEN1* is linked to deficient function of TLP18.3 under fluctuating light but the exact mechanism behind transcriptional repression of these two genes remains to be verified.

#### Cluster analysis of genes whose expression in fluctuating light requires functionality of TLP18.3

**Figure 3:**
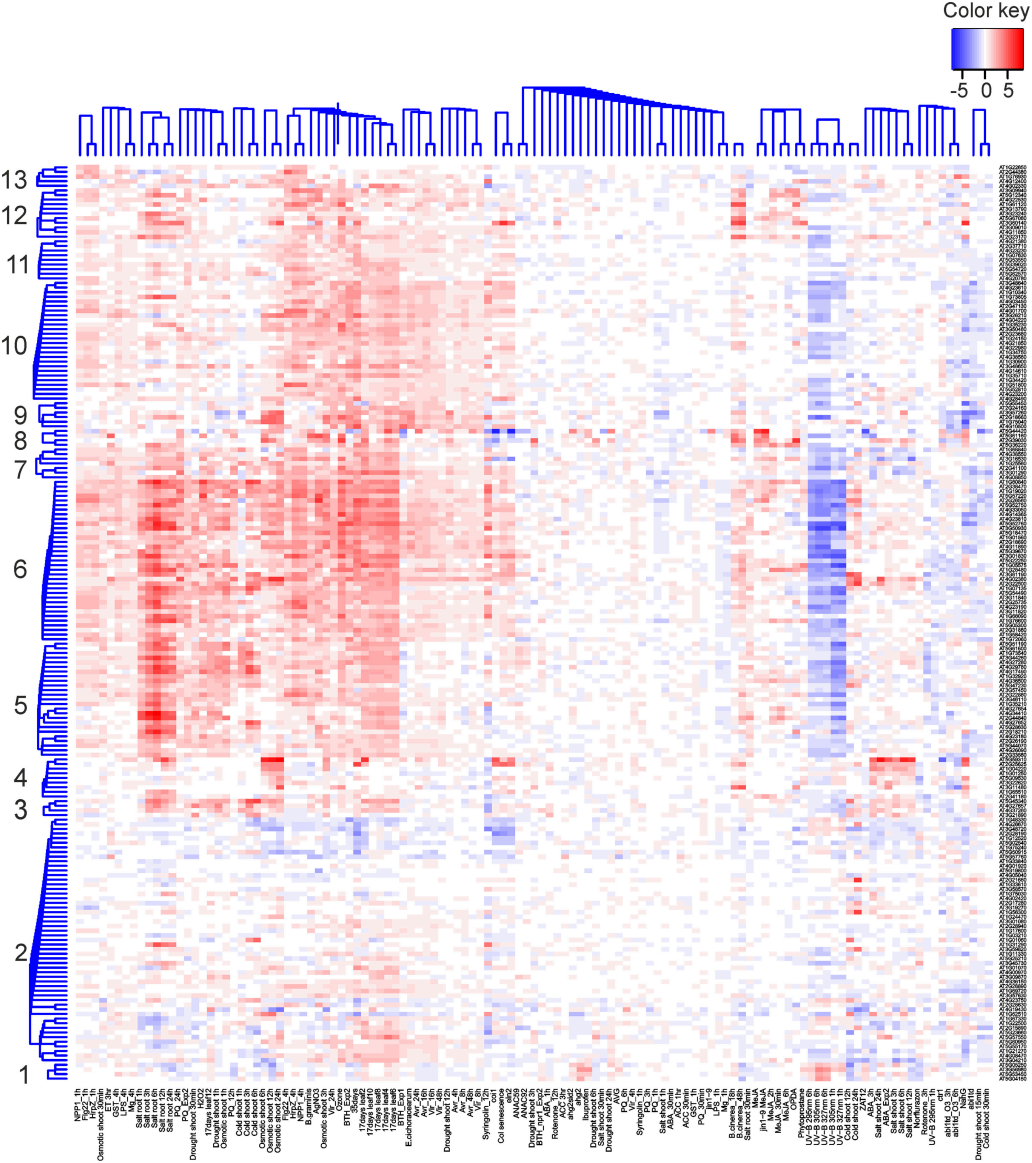
**Cluster analysis of genes differentially expressed in the wild-type but not in *tlp18.3* plants in response to fluctuating light as compared to constant growth light**. Bayesian hierarchical clustering of genes, which are significantly differentially regulated (logFC > 1) in wild type under fluctuating light as compared to constant light, is presented. Data sets used include abiotic and biotic stress experiments. Blue and red indicate decreased and increased expression as compared to untreated plants, respectively.

Finally, to shed light on gene expression changes that depend on the functionality of TLP18.3 under fluctuating light, the expression profiles of genes differentially expressed in wild type but not in *tlp18.3* upon growth under fluctuating light were clustered using publicly available datasets (Figure 3). These wild-type specific genes grouped into 13 co-expression clusters, which were further analyzed for enrichment of gene ontology categories (Supplementary Table 2). Clusters 3-13 contained genes with increased transcript abundance in different abiotic stress conditions including salinity and drought as well as methyl viologen (Paraquat; PQ) and the SA analog BTH (Figure 3). Under UV-B stress, in contrast, the expression of these genes was generally down-regulated (Figure 3). This pattern of gene expression was particularly evident within the gene clusters 5, 6, and 9, which showed significant enrichment of gene ontology categories related to plant immunity, such as “response to chitin,” “ethylene-activated signaling pathway,” or “systemic acquired resistance” (Supplementary Table 2). In wild type the genes belonging to clusters 5, 6, and 9 were generally down-regulated, showing a similar pattern to UV-B stress.

## Discussion

During the past few years evidence has been accumulated concerning the role of photosynthesis in plant immunity. Here, we have provided new insights into the linkage between light acclimation and plant immunity at the level of gene expression as well as addressed the role of the TLP18.3 protein within these processes. Chloroplasts, in addition to their main task in conversion of solar energy into chemical energy, participate in a number of other reactions like biosynthesis of amino acids, hormones, and secondary metabolites as well as cellular sensing of abiotic and biotic stress signals. Indeed, signals originating from the photosynthetic light reactions such as redox state of the electron transfer chain, accumulation of stromal metabolites as well as ROS and reactive electrophilic species are key components of chloroplast retrograde signaling (Fey et al., 2005; Piippo et al., 2006; Queval and Foyer, 2012; Szechyńska-Hebda and Karpiński, 2013; Bobik and Burch-Smith, 2015; Gollan et al., 2015). These signals respond rapidly to changes in perception of light by the two photosystems.

Here, we focused on plants grown under either constant or fluctuating light conditions for their entire life span in order to unravel how the rapid fluctuations in the growth light intensity affect the acclimation processes at the level of nuclear gene expression. In short, neither photosynthesis-related genes nor the photosynthetic protein complexes showed significant alterations as a response to fluctuating light (Figure 1, Tables 1–3). Instead, EPR spectroscopy revealed that the relative amount of functional PSI complexes was lowered in fluctuating light as compared to plants grown under constant light. Most prominently, in wild-type plants fluctuations in growth light suppressed the expression of genes related to defense reactions (Table 2A). Despite the high-light peaks of 1 min, the low-light phase is dominant in our fluctuating light setup. Hence, it is highly likely that decreased transcript abundance of the defense genes in wild-type Arabidopsis under fluctuating light is linked to shade-avoidance and is mediated by plant hormones (Vandenbussche et al., 2005; Wit et al., 2013). The experimental setup, in which the gene expression was studied from plants grown their entire life span either under constant or fluctuating light did not allow us to identify specific immune responses activated by the fluctuations in the growth light intensity. Instead, this experimental setup shed light into late stages of the plant acclimation process, in which a vast number of defense pathways were affected.

Contrary to wild type, in the *tlp18.3* mutant the alterations in the overall gene expression pattern, as a response to fluctuating light, were less evident and indeed, the *tlp18.3* plants were less capable of turning off the gene expression related to plant immunity under fluctuating light conditions (Table 2B, Figures 2, 3). It is known that the photoreceptor-derived signals activate the shade-avoidance responses and reduce the defense reactions against pathogens and pests to save resources for the growth of the plant (Ballare, 2014). Interestingly, the gene expression of two components of phytochrome-mediated light signaling, *HFR1* and *PIL1*, was shown to be altered in *tlp18.3* leaves (Table 4)*. HFR1* and *PIL1* genes are involved in transcriptional regulation pathways downstream of phytochromes, which integrate light and hormonal signals and play a role in shade-avoidance responses (Jiao et al., 2007). Of these, HFR1 also contributes to the crosstalk between light signaling and plant innate immunity (Tan et al., 2015). Based on these results, it is evident that the functionality of TLP18.3 protein modifies the light perception and/or signaling network, and possibly also the signaling related to nutrient availability (Supplementary Table 1). Allocation of resources to defense reactions in the *tlp18.3* mutant is likely associated with the lower biomass of mutant plants as compared to wild-type plants under low-light dominant fluctuating light. It should be noted that the *tlp18.3* plants also had lower biomass as compared to wild type when grown under high-light dominant fluctuating light with longer, 1 h light pulses (Sirpiö et al., 2007). It remains to be studied whether the growth phenotype of *tlp18.3* plants under high-light dominant fluctuating light originates directly from the diminished pool of active PSII complexes. Indeed, duration, frequency, and intensity of fluctuating light regimes have been shown to affect the acclimation responses in Arabidopsis (Alter et al., 2012). To that end, it would be interesting to compare how the gene expression patterns of low-light and high-light dominant fluctuating light conditions differ from each other.

Defective degradation of the D1 core protein of PSII in *tlp18.3* plants is a promising system for the search of chloroplast-derived retrograde signals which affect gene expression related to plant immunity. In line with this, low amount of the D1 degrading protease FtsH has been earlier observed to accelerate the hypersensitive reaction in tobacco (Seo et al., 2000). Recently, a link between PsbS-mediated photoprotection and pathogen resistance has also been shown to exist (Göhre et al., 2012; Johansson Jänkänp et al., 2013). Further, as the PSII repair cycle and maintenance of PSI are interconnected (Tikkanen et al., 2014), also PSI and/or PSI electron acceptors might act as a source of retrograde signaling components under fluctuating light. It should be noted that the pool of active PSII was not changed in *tlp18.3* plants as compared to wild type under low-light dominant fluctuating light (Table 1) and thus the effect might be indirect. We postulate that the compensation mechanisms activated in the *tlp18.3* mutant are likely to alter the chloroplast-derived retrograde signals. Taken together, our results demonstrate that light acclimation and plant immunity are interconnected and the proper repair cycle of PSII plays a key role in the process.

## Author contributors

SJ, JI, SK, JS, and FM contributed to acquisition, analysis, and drafting the work, while MS and EA designed the work and contributed to acquisition, analysis, and drafting the work.

## Funding

Our research was financially supported by the Academy of Finland (project numbers 272424, 271832, and 273870), TEKES LIF 40128/14, the Swedish Research Council, the Swedish Energy Agency, the Knut and Alice Wallenberg Foundation and the Initial Training Networks (ITN) CALIPSO (607607), and PHOTOCOMM (317184).

### Conflict of interest statement

The authors declare that the research was conducted in the absence of any commercial or financial relationships that could be construed as a potential conflict of interest.
